# How Much Does Movement and Location Encoding Impact Prefrontal Cortex Activity? An Algorithmic Decoding Approach in Freely Moving Rats

**DOI:** 10.1523/ENEURO.0023-18.2018

**Published:** 2018-04-27

**Authors:** Adrian J. Lindsay, Barak F. Caracheo, Jamie J. S. Grewal, Daniel Leibovitz, Jeremy K. Seamans

**Affiliations:** Djavad Mowafaghian Centre for Brain Health and Department of Psychiatry, Faculty of Medicine, University of British Columbia, Vancouver, British Columbia V6T 2B5, Canada

**Keywords:** Convolutional neural networks, ensemble encoding, random forests, recurrent neural networks

## Abstract

Specialized brain structures encode spatial locations and movements, yet there is growing evidence that this information is also represented in the rodent medial prefrontal cortex (mPFC). Disambiguating such information from the encoding of other types of task-relevant information has proven challenging. To determine the extent to which movement and location information is relevant to mPFC neurons, tetrodes were used to record neuronal activity while limb positions, poses (i.e., recurring constellations of limb positions), velocity, and spatial locations were simultaneously recorded with two cameras every 200 ms as rats freely roamed in an experimental enclosure. Regression analyses using generalized linear models revealed that more than half of the individual mPFC neurons were significantly responsive to at least one of the factors, and many were responsive to more than one. On the other hand, each factor accounted for only a very small portion of the total spike count variance of any given neuron (<20% and typically <1%). Machine learning methods were used to analyze ensemble activity and revealed that ensembles were usually superior to the sum of the best neurons in encoding movements and spatial locations. Because movement and location encoding by individual neurons was so weak, it may not be such a concern for single-neuron analyses. Yet because these weak signals were so widely distributed across the population, this information was strongly represented at the ensemble level and should be considered in population analyses.

## Significance Statement

It is often difficult to determine exactly what is driving changes in the activity of single mPFC neurons. Basic things such as movements or spatial locations can activate these neurons and interfere with the ability to extract high-order task-related correlates. Here we applied a series of powerful techniques to carefully quantify the relationship between a rat’s movements and the activity of mPFC neurons. Overall, the firing related to limb movements, poses, and spatial locations had little impact on individual neurons, yet this widely distributed information became prominent at the ensemble level. A framework where neurons participate to varying degrees in encoding all events has certain advantages that could prove useful for higher-order cognitive processing and for guiding artificial intelligence approaches.

## Introduction

Prefrontal cortex (PFC) neurons encode a wide variety of stimuli, actions, and outcomes and many neurons multiplex information across domains (Jung et al., 1998; Duncan, 2001; Lapish et al., 2008; Rigotti et al., 2013). The fact that these neurons can encode such diverse types of information greatly complicates making attributions about what a neuron might be responding to at any given time. A particularly notable example is the encoding of information during working memory tasks. While it is clear that some rat medial PFC (mPFC) neurons fire throughout the delay period of such tasks (Durstewitz et al., 2000; Laubach et al., 2000; Baeg et al., 2003; Hyman et al., 2010; Cowen et al., 2012; Wang et al., 2011; Euston et al., 2012; Horst et al., 2012), questions remain about the information represented by this activity. Delay-period activity has been linked to the prospective tracking of likely trial outcomes (Hyman et al., 2012; Myroshnychenko et al., 2017), the encoding of reward-related feedback from preceding trials (Laubach et al., 2015), or the active tracking of spatial locations, which could be highly relevant since most rat working memory tasks are spatial in nature (Jung et al., 1998; Euston and McNaughton, 2006; Laubach et al., 2015). Based on a series of careful analyses, Euston and McNaughton (2006) and Cowen and McNaughton (2007) argued that delay-period activity can also be attributed to the encoding of the paths traversed or the movements rats engage in during delay periods. These latter studies raised the broader question of how much of the activity recorded on any task is actually movement or location related.

To gain a deeper perspective on this issue, we performed a detailed video analysis of body and limb movements in rats implanted with tetrode arrays aimed at the mPFC. The goal was not to explore the absolute limits of movement or location encoding of mPFC neurons, but rather to get a realistic picture of how much movement or location encoding affects firing in a typical task situation. To approximate a task situation while avoiding overt task correlates that could interfere with the ability to cleanly extract movement or location signals, rats were trained on a simple Pavlovian conditioning task, but no tones or outcomes were delivered when the data were collected. The first analysis involved a regression through a generalized linear model (GLM) that assessed the contribution of various movement factors (the inputs) to the observed firing of individual neurons (the outputs). Because movement information may not be completely contained in the firing of individual mPFC neurons taken one at a time, we also performed several different types of ensemble analyses. For these analyses, the firing rates of all neurons were used to predict the rat’s spatial location, the position of individual limbs, or constellations of limb positions (i.e., postures or poses). Because it was unlikely that all neurons contributed equally to all of these factors, the random forest (RF) algorithm was employed because it was able to iteratively consider random subsets of neurons to select the best candidates based on a measure of mutual information between firing and a particular factor. Several neural network (NN) models were also used that considered relationships between small groupings of neurons across a range of time scales (temporal lags and leads). Results indicated that spatial location and movement information was distributed across the population but accounted for at best <20% of firing rate variance in those neurons maximally sensitive to a particular factor. Ensembles, however, performed better, especially in terms of spatial decoding.

## Materials and Methods

### Experimental subjects and operant chamber

Four male Long-Evans rats (Charles River Laboratories) weighing between 400 and 470 g were used. They were housed in an inverted 12-h day/12-h night cycle and were food-restricted to 90% of their free-feeding weight but given unlimited access to water for the duration of the experiment. All procedures were conducted in accordance with the Canadian Council of Animal Care and approved by the Animal Care Committee of the University of British Columbia. Recording sessions took place inside a custom-made behavioral chamber (30 × 25 × 60 cm) built for a Pavlovian conditioning task (Caracheo et al., 2015). However, the presented data were recorded while the rats moved freely in the chamber in the pretask period in the absence of any tones or outcomes.

### Surgery and electrophysiology data acquisition

**Figure 1.**
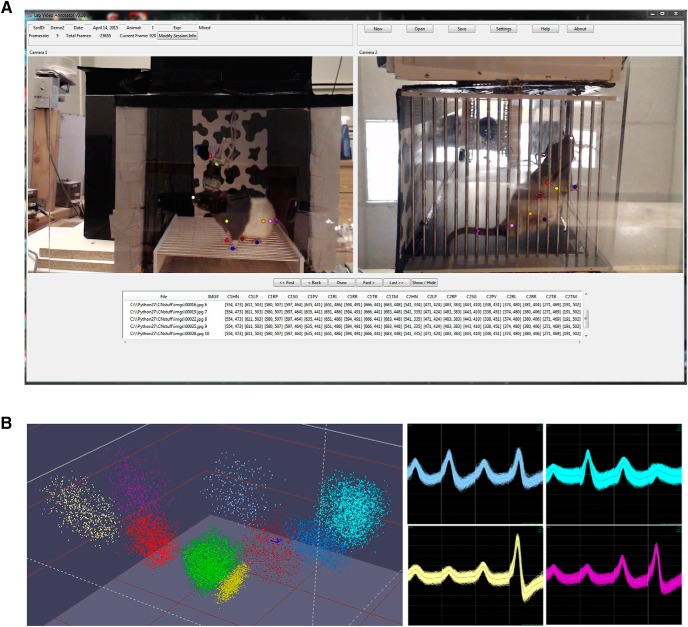
***A***, Multicamera video annotation. A frame from the annotation set showing a rat moving freely in the operant chamber. The colored dots on both views serve as markers for tracked limbs and body parts. The annotations from both camera views are combined to produce an accurate 3D location of each tracked body part for each frame. The user can select the camera that provides the most accurate view of a limb, and the *xyz* coordinates of the limb are simultaneously captured in both data streams. This software does not automatically detect these points, but they are set by the user in each frame. ***B***, Spike sorting based on waveforms recorded from a single tetrode. Left, clouds of spiking events from different putative single units. The dimensions of the space are relative peak-to-valley distances across three of the four tetrode wires. Right, four of the wave form collections corresponding to the four similarly colored clouds shown at left. Each wave form comprises four perspectives of the unit’s spikes from the four wires of the tetrode.

Rats were surgically implanted with a custom-built 16-tetrode hyperdrive array (Hyman et al., 2012; Ma et al., 2016). Each rat was anesthetized under isoflurane gas, the skull was surgically exposed, and a 4 × 3-mm hole was drilled. The dura was removed to expose the brain around coordinates 3.0 mm from bregma and ±0.5 mm from the midline. The tetrode microdrive implant was positioned over the area and fixed to the skull with 11 skull screws and dental acrylic. Two additional screws were used as ground wires and were placed in the posterior skull. Tetrodes were lowered ∼1000 μm on the day of surgery, and then the rats were given 1–2 wk of recovery. Tetrodes were advanced up to 1000 μm more before the first recording session. Each day tetrode drives were turned between 20 and 50 μm to maximize the units recorded and obtain different populations from days prior. Based on tetrode advancement records, the positions were estimated to have been in the medial wall, within the anterior cingulate cortex (ACC) up to the border of the prelimbic (PL) cortex. Tetrodes were attached to EIB_36_TT boards, plugged into two HS-36 headstages, and connected via tether cables to a Digital Lynx 64-channel system and then to a PC workstation. Electrophysiological data and behavioral events were captured using Cheetah 5.0. Files were exported into Offline Sorter (Plexon) and manually sorted based on 3D projections of wave form peaks, valleys, and principal components (Fig. 1*B*). Once cells had been sorted, spike data were exported to Neuroexplorer 4 (Nex Technologies) and then to Matlab (Mathworks) for further analysis. When the experiments ended, rats were perfused, and brains were collected and sliced on a cryostat. Slices were mounted on slides and viewed under a microscope to confirm the anatomic locations of tetrode tracts.

### Software and acquisition of video data

A camera was placed horizontally to capture a side-on view of the chamber, and another camera was placed below to capture a bottom-up view. The two video streams were synchronized, coregistered, and imported into a custom, purpose-built Python package for video annotation. This software allowed us to annotate body and limb positions on a frame-by-frame basis for both camera views (Fig. 1*A*). The software synchronized the frame rate of the video with that of the neural data (200 ms) and allowed the user to position the points in each frame. All point placements were done by hand and did not involve automation of any kind. The camera that provided the best view of a limb was used to score the limb position in each frame. The central point of the skeleton was set to the center point of the rat’s body. The *x-y-z* location of this point gave the spatial location of the animal at each time bin. Changes in the *x-y-z* coordinates across time bins were used to calculate the animal’s velocity. The positions of nine body parts (head, midshoulder, front left limb, front right limb, pelvis, rear left limb, rear right limb, base of the tail, midpoint of tail) were calculated relative to the center point of the body. Because the two camera views were coregistered, the body positions could be collapsed to nine values/time bin. Therefore, each time bin was associated with a spatial location (the *xyz* coordinate of the center of mass), one instantaneous velocity value, and nine body positions (relative to center of mass) that were temporally aligned with an *N*-item spike count vector (*N* = number of neurons). This alignment was set by paired pulses to the video and electrophysiology recording rigs, matching the timing to within 1 ms.

### Data analyses

The annotated positions were broken down into three data sets for each session: (1) individual body positions as defined by the *xyz* coordinates relative to the center of mass of the animal (for analysis as single factors, these relative coordinates were collapsed to a single distance measure); (2) the velocity of the animal at each point in time; and (3) the location in 3D space of the animal within the enclosure.

#### Generalized linear model

The GLM modeled the firing response as a Poisson sequence with a logarithmic link-function as follows: predictors η were linear combinations of parameters (η = *X*β), linked to the mean μ of the output by the link function (*X*β = In(μ)). Regressions that showed a significant relationship between a factor and firing rate (*p* ≤ 0.01) denoted the neuron as responsive to that factor. The GLM was run on each factor independently for each neuron.

#### Random forest

**Figure 2.**
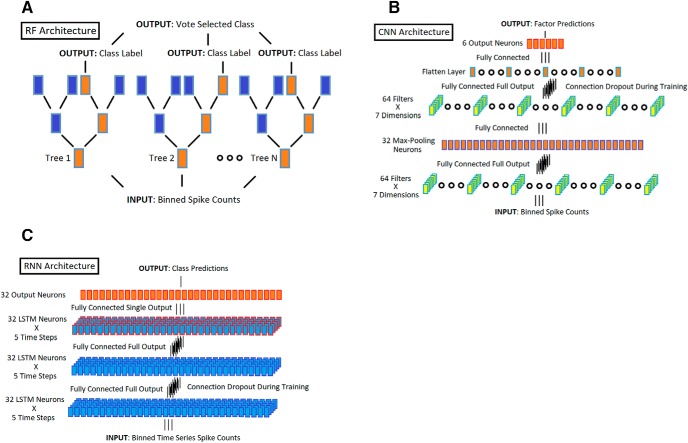
Model architectures. ***A***, RF architecture. Diagrammatic representation of the RF utilized for ensemble regression/classifications. Input in the form of binned spike counts was introduced at the bottom of the trees and progressed through each tree to the top-layer output. The output layer consisted of the class votes of the all the individual trees in the forest. During training, the trees were constructed decision layer by decision layer. At each layer of each tree, a random subset of the inputs (neurons) was selected. A single input was then selected from this group based on maximal mutual information with the output and used to define a single decision. This process continued until the classes were uniquely separated in each tree. The validation error for training was done by out-of-bag testing on the constructed trees. ***B***, CNN architecture. Diagrammatic representation of the CNN utilized for ensemble regression on body part position. Input in the form of binned spike counts entered the network at the bottom and progressed through the computational layers to the output layer at the top. The network contained two convolutional layers (yellow boxes) of 64 filters, each of which was 4 to 7 × 1 units in size. The output of the first of these layers is condensed (orange boxes with purple edges) by maximum pooling before being passed to the second convolutional layer. This output layer is flattened (orange boxes with blue edges) before being passed to a final layer of fully connected output units which provide the predicted body/limb position signals. During training, the output of the convolutional layers was subject to connection dropout to reduce overfitting and improve performance. ***C***, RNN architecture. Diagrammatic representation of the RNN utilized for ensemble categorization of spatial locations. Input, in the form of a fixed length time series of binned spike counts (5 × 200-ms bins, or 1 s in length), entered the network at the bottom and progressed through the computational layers to the output layer at the top. The network contained three LSTM recurrent layers, each with 32 units. The first 2 layers (blue boxes with purple outlines) are fully connected and passed the full 5 time-steps on to the next layer. The third layer (blue boxes with red outlines) only passed the last step in the time series on to the final output of the layer, which consisted of 32 fully connected units with Softmax activation to provide the predicted location. During training, the connections between the LSTM layers were subject to dropout to reduce overfitting and improve performance.

RF analysis was conducted using the Python Sci-Kit Learn package (Pedregosa et al., 2011). The RFs were tuned for forest size and split size using “out-of-bag” (OOB) error across all sessions. OOB error, the error rate on OOB samples, is calculated as follows. Each individual tree in the RF is trained on a subset of the total training set. Samples from outside this subset, or bag, which are unseen during training by that tree, are used to validate each tree. The average across all trees is referred to as OOB error. The RFs were trained on a per-session basis using a normalized, balanced (by class), random subset of time bins (by cross-validation) and then tested by prediction on the remainder of the time bins. The parameters with the best average performance for each factor across all sessions were reported. The RF architecture schematic is shown in Fig. 2*A*.

#### Neural networks

The networks were coded using the Keras package for Python (Chollet et al., 2015), which used Theano (Al-Rfou et al., 2016) to perform tensor operations. Our networks were run with GPU parallelization using nVidia’s CUDANN package (Nickolls et al., 2008). A multilayer convolutional neural network (CNN) was used to evaluate the regression between the multidimensional input (neuron spike count data) and output (relative limb positions) space, as described in Fig. 2*B*. The network comprised several convolution layers of rectified linear (relu) units, with a mean-squared error loss [between predicted (y-hat) and the actual (y), limb position as follows: E=12NΣi=1N‖y^l−yi‖2). The network was trained on a per-session basis using a normalized, random subset (80%) of time bins with 10% held out as a validation set and then tested by prediction on the remaining 10% to generate the reported results. The recurrent neural network (RNN; Graves, 2016) was used for spatial position encoding. After structural testing and optimization, the multilayer RNN evaluated the relationship between time series of the multidimensional input (the binned firing rates) and output (the spatial location factor) space, as described in Fig. 2*C*. It considered not only the current time bin but the four to six time bins that preceded or followed it to better inform its decisions about the spatial location of the rat. The network comprised layers of long-short-term memory units (LSTMs), a modification on the basic recurrent structure that minimized the impact of numerical instability during back-propagation (Hochreiter and Schmidhuber, 1997; Gers et al., 2000). The output layer was a softmax layer (LeCun et al., 1998; Bishop, 2006), trained using categorical cross-entropy as the loss function (this measured the cross-entropy between two distributions; an estimated distribution, *q*, and the true distribution, *p*, computed as follows: *H*(*p*, *q*) = −Σ*_x_p*(*x*)log(*q*(*x*)). As above, the network was trained on a per-session basis using a normalized, class-balanced, random subset of time bins, with 10% held out as a validation set and was then tested by prediction on the remaining 10% to generate the reported results.

One major concern with unconstrained predictors with many parameters like NNs is the tendency to overfit, particularly on small data sets like the one considered here (Hastie et al., 2009). We utilized an effective method to curb this overfitting by preventing complex co-adaptations, or paired relationships between units in the network from forming with a technique called dropout, whereby connections between individual units were dropped with some specified probability during training (Srivastava et al., 2014; Gal and Ghahramani, 2016). We formulated the problem as a categorical prediction to further combat overfitting, compensate for uneven distributions of locations in the data, and disambiguate location from relative distance (each location was technically treated as equidistant from every other location, so that the algorithm learned the true location correspondence, as opposed to some amalgamation of averaged distances). The enclosure was divided into two 4 × 4 grids stacked on top of one another. The position for each time bin was set as the cube containing the rat’s center of mass. The training set data were always balanced for cube occupancies. In all cases, our NN were trained and evaluated using cross-fold validation to combat unintended effects on performance due to evolving distributions in the data. Reported results are averaged across folds for a given session.

### Code accessibility

The code described in the paper is freely available online at the following URLs: https://github.com/Loken85/Lab_Video_Annotator and https://github.com/Loken85/ephys_ML_repo.

## Results

Fig. 1*A* provides a screenshot of the custom-written application used to track spatial locations and body positions throughout each of the sessions. The central point of the rat’s body (the *xyz* coordinate of the midpoint between shoulders and pelvis) was taken as the spatial location of the animal at each time bin. Changes in this point across time bins were used to calculate the animal’s velocity. The positions of nine body parts (head, midshoulder, front left limb, front right limb, pelvis, rear left limb, rear right limb, base of the tail, midpoint of tail) were defined as the distance between the *xyz* coordinates of the part relative to the *xyz* coordinates of the center point of the body in each time bin. Consequently, each time bin was associated with one spatial location, one instantaneous velocity value, and nine body position values that were temporally aligned with an *N*-item spike count vector (*N* = number of neurons).

### Single-unit analysis

**Figure 3.**
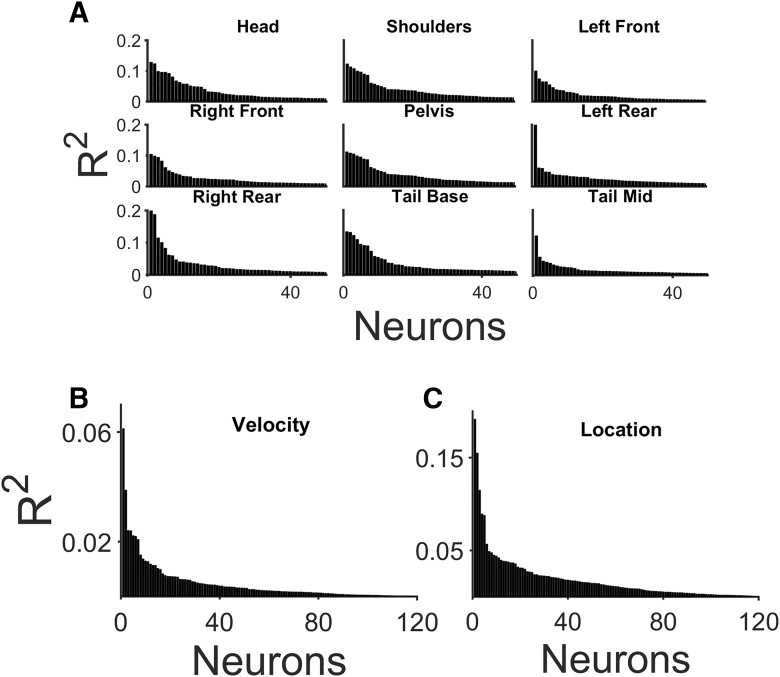
Relationship between firing and the rats’ movement and spatial locations. The coefficient of determination (*R* ^2^) between the firing rate of individual neurons and each of the body position factors (***A***), the general movement/velocity factor (***B***), and the spatial location factor (***C***). Only the *R* ^2^ values of the top 50 (***A***) or top 120 (***B***, ***C***) neurons significant (*p* < 0.01) on a given factor are plotted. Total significant neurons of each factor were head = 181, shoulders = 193, left front limb = 170, right front limb = 170, pelvis = 194, left rear limb = 179, right rear limb = 157, tail base = 173, tail mid = 124.

**Figure 4.**
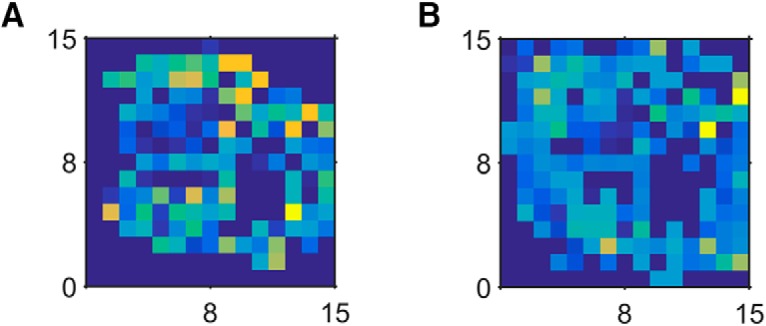
Single neuron location mappings. ***A***, ***B***, Normalized heatmaps of firing of two single neurons mapped to quantized 2D (*x*-*y*) locations. The color in this case represents the relative firing of the neuron over the space. The box was divided into a 15 × 15 grid, with each square of the grid measuring 2 cm (depth) × 1.67 cm (width).

The database for the present study was derived from 8 recording sessions in 4 rats and contained a total of 492 neurons. The first analysis involved a GLM. For each neuron, the input, or predictors to this GLM, were the animal’s current spatial location, instantaneous velocity, or limb positions, while the spike counts made up the output. Across all factors, on average, 38% of the neurons were significant on any single factor. 61% of the neurons attained significance on at least one body position factor (Fig. 3*A*). However, for those neurons that showed significant responses, the *R* ^2^ between the model factor and the firing rate vector was relatively low (<0.1; Fig 3*A*), such that any of these factors accounted for at most <20% of a neuron’s sessionwide firing rate variance. Possibly owing to the small enclosure size, only 26% of the neurons were found to be significant on the velocity factor, and in none of these cases were high *R* ^2^ values observed (Fig. 3*B*). As a result, the velocity factor was not analyzed further. In total, 134 neurons (27%) were significant on the spatial location factor, but individually none of these neurons were particularly good at accurately encoding spatial location, and *R* ^2^ values >0.1 were found in only a few cases (Fig. 3*C*). Fig. 4 shows the firing maps of two neurons with relatively high *R* ^2^ values (0.15 and 0.08) on the spatial location factor. Despite attaining statistical significance, their firing was diffuse but nevertheless variable across the enclosure.

**Figure 5.**
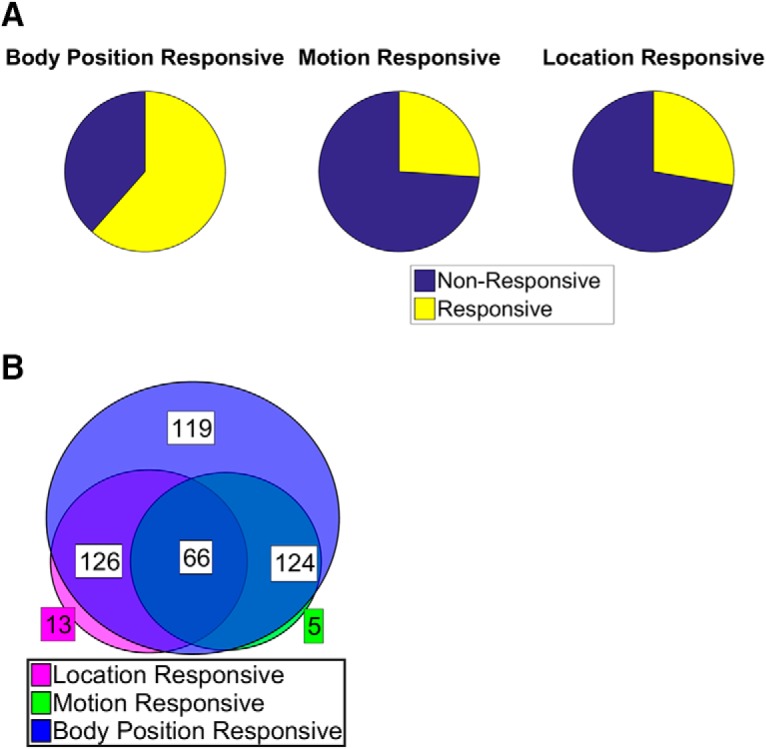
Proportions of neurons significant on a given movement/position factor. ***A***, The percentages of neurons (of a total of 492) that had a significant response (*p* < 0.01) to movement of a given body part (left), overall velocity (middle), or spatial location (right). ***B***, Venn diagram of neurons responsive to the three categories of factors and their overlap. Blue, number of neurons responsive to at least one of the body position factors; green, number of neurons responsive to velocity; magenta, numbers of neurons responsive to spatial location.

Most neurons tended to be multiresponsive, as 42% of limb-responsive neurons were responsive to spatial location and 41% to body movement, while 13% were responsive to all three factors (Fig. 5). This high degree of overlap is somewhat misleading, as the factors were not independent in that they provided different perspectives on the same moving object. This multi-collinearity could not be avoided and was the reason the model was run independently for each factor. As a consequence, it was difficult to parse the relative contributions of the factors to the overall firing rates of the neurons.

### Ensemble encoding of individual limb positions

**Figure 6.**
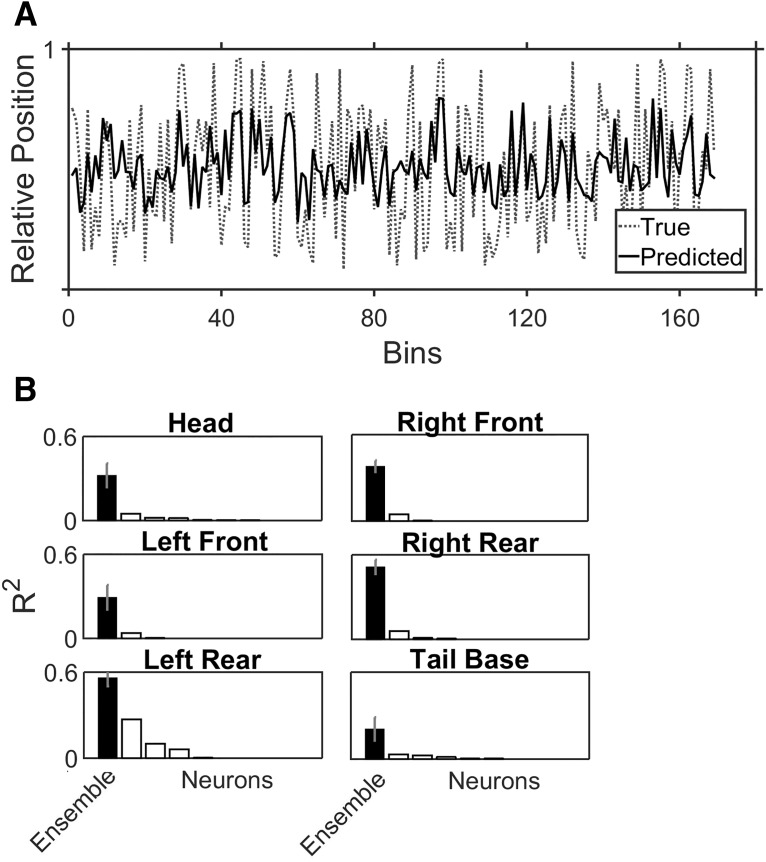
Ensemble versus single-neuron encoding of body positions. ***A***, An example of the actual head position across 180 frames/time bins (36 s; dotted gray line) versus the predicted head position (black line) derived from the RF. ***B***, The overall average (and SD) *R* ^2^ values between the RF-predicted and the actual body/limb positions are plotted alongside the ordered single neuron *R* ^2^ (white bars) values from GLMs run on a single session.

The ensemble analysis of body movements was formulated as a regression problem using the RF algorithm with the binned spike counts of all neurons as inputs and the body/limb position factors as outputs. To facilitate comparisons with the single-neuron data, encoding performance was evaluated by calculating an ensemble *R* ^2^ relative to the model factors. The RF did a reasonable job of predicting the animal’s relative head (Fig. 6*A*) or limb positions, and *R* ^2^ values for the ensembles were relatively high on most factors (Fig. 6*B*), with an overall average of 0.36. Direct comparisons to single units from the same session (as shown in Fig. 6*B*) highlighted the superiority of the ensemble, as *R* ^2^ values derived from the RF were usually higher than the sum of the *R* ^2^ values derived from the GLMs of all the significant individual neurons. While the ensembles were better overall, part of this improvement stemmed from the fact that the RF selectively considered only the informative portion of a given neuron’s activity with regard to a particular factor.

### Ensemble encoding of constellations of limb positions

The single-neuron and ensemble analyses considered each body/limb position factor in isolation, yet it seemed unlikely that any mPFC neuron would be so precisely tuned to the position of a single limb. Therefore, we considered whether mPFC ensembles encoded poses or constellations of body/limb positions. As an example, rearing would be a well-known pose created by a recurring constellation of relative limb position values; however, we refrained from providing labels for arbitrary possible poses (limb positions were continuous variables, yielding infinite possible arrangements). The RF was again used for this analysis but in a slightly different way. The forests first learned the relationships between ensemble activity and possible poses and were then used to predict poses (i.e., provide a probability that the limbs were collectively in certain positions) in unseen data based only on ensemble activity. The mean squared error (MSE) was used to evaluate the difference between the body/limb positions that were predicted from ensemble activity versus those that were observed in the test data. In addition to the RF, a CNN (Fig. 2*B*) was also used that attempted to find the relationships between distinct poses and unique activity patterns in sets of neurons taken four to seven at a time. The CNN in essence treated each neuron as a partial feature detector and learned the activity patterns in local groups of these feature detectors that were predictive of constellations of body/limb positions or poses. It should be noted that these algorithms did not quantify or categorize these constellations as a part of this process. As such, no attempt was made here to further investigate specific poses.

**Figure 7.**
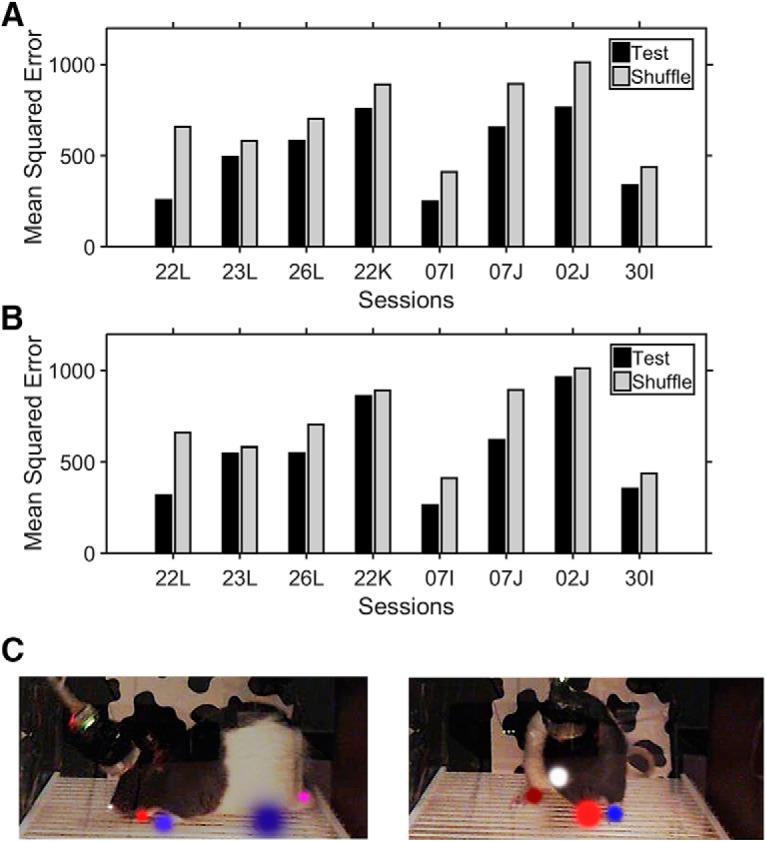
Predictions of body positions. ***A***, The overall mean squared error (MSE) between the true and predicted positions of all six pose components used in the RF analyses. Black bars are test MSEs, gray bars are the MSEs when the firing rate matrix was shuffled independently of the output poses. ***B***, Same as ***A***, but for the CNN. ***C***, Two images of the animal taken from single video frames in session 07J were overlaid by colored circles representing the MSE in the positions of the body/limbs positions as predicted from the RF analyses. Each circle is centered at the actual position of the body part as originally scored using the software shown in Fig. 1, while the radii of the circles denote the MSE in the RF-predicted positions (dot colors: head, white; front right paw, red; front left paw, blue; rear right paw, dark red; rear left paw, dark blue; tail, pink). The overall average MSE across all body parts depicted in the images were 69.7 (left) and 122.4 (right). Only the MSE spheres for those body parts that can be clearly seen in the example images are depicted here.

We found that both the RF and CNN were able to provide reasonable predictions of learned poses in the unseen test data. Despite some variance across the sessions, the average MSE was significantly below what was obtained when the time bins were shuffled (Fig. 7*A*, *B*) or when the actual spike counts for each neuron were substituted with spike counts generated by random Poisson processes with identical means (not shown). To provide context for these MSE values, Fig. 7*C* (right and left) shows examples of the degree to which the poses predicted by the RF differed from the actual poses. In these examples, the size of the spheres indicates the MSE of the predicted body/limb positions depicted.

### Ensemble encoding of spatial position

**Figure 8.**
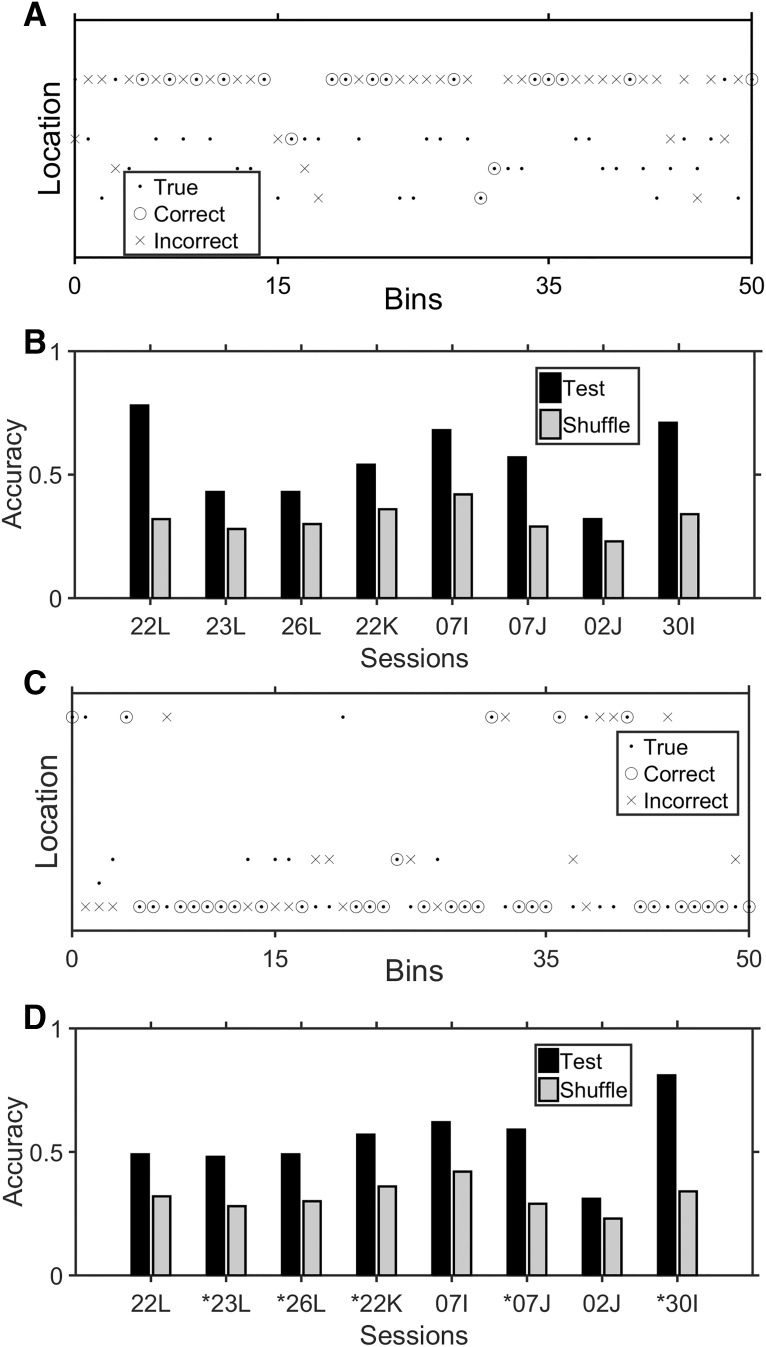
Predictions of spatial locations. ***A***, Spatial location predictions generated by the RF for unseen test data across a 10-s period (50 video frames/time bins). The true location is indicated by a black dot, the circles indicate correct location predictions, and X’s indicate incorrect location predictions. This figure contains only the subset of 4 locations (i.e. cubes, ordered on the *y*-axis) the animal happened to visit during the chosen time period. ***B***, The location accuracy per session for the RF classifiers. Black bars, accuracy of predictions for the test data; gray bars, accuracy of predictions when position assignments were shuffled independently of the firing rate matrix. ***C***, ***D***, Same as ***A***, ***B***, but for the RNN.

We used a similar approach to try to predict the location of the animal within the enclosure. Again, we felt that it would be unrealistic to expect that the ensembles could predict exact locations on the scale of individual pixels, so the operant chamber was divided into 32 cubes (two stacked 4 × 4 grids), allowing us to frame location prediction as a categorical problem. As in the analyses above, we first tried the RF (Fig. 8*A*) and found that it could provide reasonable predictions about the spatial location of the rat based on ensemble activity. While the quality of spatial location encoding was variable both within (i.e., depending on which subsets of time bins were used) and between sessions, accuracy was always higher compared to chance performance assessed by shuffling of the spatial location assignments in each time bin of the original data (Fig. 8*B*). The ensembles exhibited a nearly 50% accuracy rate for determining the location of the rat, although the chance rate was on average 1/32 or 3%. It should be emphasized that these accuracy measurements were conservative in the sense that they were calculated from the strict maximum of the probabilistic output, and instances where the prediction probabilities were split across two or more cubes were counted as errors. Furthermore, all errors were equal regardless of whether the predicted cube was adjacent to the actual cube or on the other size of the chamber. The CNN performed statistically no better than the RF, and therefore the results from the CNN were not shown.

Previously, Euston and McNaughton (2006) found that some mPFC neurons were sensitive to the trajectory the rat took through a task enclosure. For this reason, we also considered whether the location predictions could be improved by incorporating trajectory information as the rat entered or exited a specific location. Based on the observation that the ensembles carry information about the rat’s spatial location (Fig. 8*A*), we reasoned that trajectory information should be contained in the spike count time series. A multi-layer RNN (Graves, 2016; Fig. 2*C*) was chosen for this purpose that evaluated the relationship between time series of multidimensional inputs (the binned spike counts) and outputs (the spatial locations) across 1-s epochs (i.e., 5 × 200-ms time steps). During training, spike counts from a given time bin plus the 4 time-bins that flanked it were used to update the weights between the units in the RNN. An example of location output prediction for the RNN is shown in Fig. 8*C*, while accuracy measures for each session are shown in Fig. 8*D*. Similar to the RF, the predictions made by the RNN were probabilistic across possible locations, so results were conservative measures of maximum probabilities. The predictions were significantly better for the RNN than the RF in 5 of the sessions (* in Fig. 8*D*). This improvement was likely realized because the RNN learned which cubes would likely be visited based on the evolution of ensemble activity through time, thereby constraining the probable output space.

## Discussion

In the present study, we precisely tracked nine points on a freely moving rat’s body every 200 ms in the absence of an overt task and then quantified the relationship between this body position data and the firing of multiple single neurons recorded simultaneously in the mPFC. While a significant relationship was found between firing and the rat’s movements or spatial locations, the relationship was uniformly quite weak for individual neurons. The ensemble analyses exploited the signals of the best neurons and as a result returned better results. The superior performance of the RNN over the other ensemble algorithms in predicting spatial location indicated that additional information was contained in the time series of firing activity that evolved as the rat moved through the enclosure.

Jung et al. (1998) performed the first detailed study of mPFC correlates and described >20 different movement correlates as well as the broad spatial tuning properties of these neurons. Euston and McNaughton (2006) and Cowen and McNaughton (2007) later highlighted that movement/location signals and putative cognitive signals can be difficult to disambiguate. Expanding on this work, we sought to provide a detailed assessment of the degree to which movements or locations affect the firing of mPFC neurons in a typical task setting. It should be noted that the apparatus and environment were not designed specifically to optimize movement or location encoding, and a more repetitive task, performed over many days, would likely more strongly entrain neurons. The enclosure was also quite small, and it is possible that exploration of a larger or novel enclosure would have evoked stronger neural responses. Furthermore, the recordings were performed outside the context of an overt task (but in a typical task environment) so as to preclude nonmovement correlates from interfering with the analysis. While there are obvious advantages to this approach, movement signaling in mPFC is highly plastic, and action correlates can be altered if the action leads to a motivationally relevant outcome (Kennerley et al., 2009; Ma et al., 2014). As a result, the present results do not speak to the absolute strength of movement or spatial encoding in mPFC neurons but rather provide a picture of how strongly this information is encoded under baseline conditions when rats are placed in an operant chamber used for prior behavioral training. We found that although a large proportion of neurons showed significant responses to movement of a single body part, their individual contributions were small, and even the activity of the best single neurons was insufficient to accurately decode the position of a single limb. These data therefore suggest that under baseline conditions, most mPFC neurons are weakly responsive to a wide range of proprioceptive, posture, and position information. Any component of these diffuse representations could conceivably become strengthened depending on the prevailing task demands.

Ensembles generated more complete descriptions of movement and location encoding than single neurons, although the level of improvement depends to some extent on how one sets up the analyses. One issue is whether to treat all neurons equally or weigh certain neurons (or some portion of the firing range of certain neurons) more than others. In the present study, this selection process was automated in an informed manner by the RF algorithm. Generally speaking, an RF is a collection of decision trees that decides on an output using a process of aggregate voting and selection of maximum probability (Breiman, 2001). RFs have an advantage in unconstrained problem spaces in that they are resistant to overfitting, because the estimate quickly approaches the expectation of the distribution as the number of estimators (trees) grows (Hastie et al., 2009). Each branching point of a tree in the forest considers only a randomly selected subset of the input dimensions (in this case neurons) and selects the candidates from that subset based on a measure of mutual information with the output (in this case segments of a limb position or spatial location vector). Therefore, each decision tree could be viewed as a weighting of neurons on a given factor (or portion of a given factor), with the forest providing an aggregate of single neuron mappings to all the model factors. In the GLM analyses, the firing rate of each neuron was correlated (via a link function) to a limb position vector across all time bins. By contrast, the RF created trees with neurons responsive to portions of a limb position vector, and these trees biased the forest consensus, which in turn helped to make the RF predictions more accurate.

Another advantage of the ensemble analysis was that it allowed us to search for the encoding of constellations of co-occurring limb positions that we referred to as poses. The CNN (Dumoulin and Visin, 2016) searched for functional relationships within small groups of neurons, taken four to seven at a time. Each neuron was treated as a partial feature detector, and patterns in local groups of feature detectors encoded specific portions of a given pose. The complete pose was encoded by the combined information across many such subgroups of neurons. Although the relationship between neural activity and the poses were statistically significant, both of our predictive algorithms work agnostically of predefined constellations of body positions, and as such, the output or pose space does not necessarily yield separation into meaningful or behaviorally relevant and recurring poses, such as grooming, rearing, etc.

Ensembles were also superior to single neurons for spatial decoding. While the RF performed well, adding complexity to the model with a CNN did not result in significant improvements. However, adding the additional temporal dimension made the RNN superior to single neurons, the RF, and the CNN. This was likely because the animals were moving from one location to another, and by using the time series information, the RNN limited the range of possible future or past locations on which to base its predictions. Interestingly, improvements from temporal signals (Karpathy et al., 2015) were not observed in the decoding of pose (the RNN did not perform better than the RF or CNN), as one might expect. This type of time series–based encoding would complement the real-time tracking of movement trajectories by individual mPFC neurons as identified by Euston and McNaughton (2006). The mPFC could in theory use knowledge about where the rat has been, where it is going, and the path taken between these two points to inform ongoing decisions.

The finding that ensembles performed better than the best single neurons is not surprising and is consistent with past studies conducted on neurons from various brain areas (Foldiak and Young, 1995; Riehle et al., 1997; Shadlen and Newsome, 1998; Laubach et al., 2000; Carmena et al., 2005). One reason the individual neurons performed poorly in the present study was their inherent unreliability in that they did not respond the same way every time a limb was in a particular position. While this is disadvantageous when considering each neuron in isolation, it may be beneficial for ensemble encoding. We have observed that a given mPFC neuron can respond to a certain task element on one trial, only to respond to another task element or not at all on the next trial (Ma et al., 2016). But the nonresponsive trials tend not to be shared across the population, so that when one neuron drops out another fills in, thereby maintaining a constant level of overall responsivity in the network (Ma et al., 2014, 2016). In the field of computer science, the phenomenon of dropout has proven to be an advantageous feature for neural network–based machine learning algorithms. In this case, dropout occurs when individual connections are eliminated with some probability during the training of networks, as was the case for our CNNs and RNNs. This dropout helps to suppress the formation of strict dependencies between individual units and a particular output feature. This prevents overfitting and overspecialization (Srivastava et al., 2014; Gal and Ghahramani, 2016) and makes the network better able to generalize across tasks. Although direct comparisons between biological brains and “neural” network models should be made with caution, it may be that frontal cortex networks use their inherent unreliability for similar purposes. Specifically, multiresponsivity coupled with high trial-to-trial variability would ensure that the neurons do not become entrained to specific events. On the other hand, because the variability or dropout is not synchronized across the population, the ensembles always maintain an accurate representation through time. As discussed above, most neurons were minimally responsive to most of the factors, and this may have extended to all neurons if the recording periods had been extended or the significance criteria had been slightly relaxed. Since past studies have reported an almost innumerable array of other frontal cortex neuron correlates, it may be that all frontal cortex neurons are at least minimally responsive to all events. Like the superior performance of the RF algorithm, frontal cortex ensembles may produce coherent and consistent representations via an aggregate voting process across groups of highly variable and unreliable neurons. These properties may be what gives the frontal cortex the flexibility required to respond to both the similarities and differences inherent in complex, ever-changing environments. Endowing artificial neural networks with similar properties could conceivably expand their flexibility and functionality as well.

Lab Video Annotator Repository: https://github.com/Loken85/Lab_Video_Annotator

Electrophysiology Machine Learning Repository: https://github.com/Loken85/ephys_ML_repo

10.1523/ENEURO.0023-18.2018.ed1 Extended Data 1 Lab video annotator. Compressed code package for the lab video annotator used to annotate video files. Download Extended Data 1, ZIP file.

10.1523/ENEURO.0023-18.2018.ed2 Extended Data 2 Electrophysiology machine learning repository. Compressed code package for the algorithms used in data analysis. Download Extended Data 2, ZIP file.

## Synthesis

Reviewing Editor: Li Li, New York University Shanghai Decisions are customarily a result of the Reviewing Editor and the peer reviewers coming together and discussing their recommendations until a consensus is reached. When revisions are invited, a fact-based synthesis statement explaining their decision and outlining what is needed to prepare a revision will be listed below. The following reviewer(s) agreed to reveal their identity: Jeffrey Erlich, Aaron Wilber

Both reviewers think the ms is interesting and addressed an important question in the field. Both reviewers also have some concerns that should be addressed before the ms can be accepted for publication. The reviewers' comments are detailed below:

Reviewer 1

1. It would be helpful if the authors included a link to a code repository so others could replicate their analyses and learn, from their example, how to use modern machine learning techniques.

2. The authors used a pretty small box for this study. I think they should mention that as a potential limitation in the discussion. Perhaps in a larger arena, neurons would more stringly encode location.

3. “On average, 38% of the total neurons were significant on any single factor while 61% of the neurons were significant on at least one factor.” What is the difference between ‘any single factor’ vs. ‘at least one factor’? This is confusing. Oh... I think you mean “38% encoded one (and only one) factor. 61% encoded one or more factors”

4. Give titles to Fig2 B and C (like in 2A)

5. Figure 6&7. Presumably each train-validation-test sampling was done many times, so there can (and should) be error bars in A and B to give a sense of whether test is different from shuffle. Ideally, the order of the bars would be validation, test, shuffle, since the meaningful comparison is test vs. shuffle.

Reviewer 2

Major:

A main point is that single units provide poorer information about posture than the ensemble of single units. It would be useful to include some metric of single unit isolation quality, either a representative example, or a metric reported in the text, to demonstrate that poor single unit isolation is not potentially contributing to this result.

Minor:

1. Velocity coding was not observed, and given that the dimensionality of spatial locations was very limited (size of rat vs chamber), in my opinion, a focus on posture alone would be more appropriate. Though, I am not opposed to including this information and letting readers decide for themselves.

2. If spatial analyses are retained, a 2D spatial firing rate map and/or paired plot would be much more useful, since 2D plots are standard for the field and thus more easily compared to published literature.

3. In the abstract ‘tracked with two cameras’ implies automated tracking, maybe ‘marked’?

4. Significance statement has a typo ‘to vary degrees’.

5. Discussion, end of second paragraph, ‘proprioceptive’? I did not see evidence that this coding represents proprioception vs other possibilities, so I would recommend sticking with ‘posture’ or similar terminology.

6. I am a confused about why the specific poses were never discussed, a sentence further clarifying this would be useful. A simple description of a few example poses? Or were the significant poses not easily describable? If so that would also be useful information to convey.

7. Fig. 8 should actually be Fig. 2 since it was first referenced between the current Figs. 1 and 2. Further, several figures were referenced in part but never in whole in the text. For example a reference to fig Xb, but no reference was made to the same fig. a.

8. The term ‘out-of-bag’ is jargon and as far as I could tell is never defined or elaborated on in the text.
