# Supplementary figures and images for: How Much Does Movement and Location Encoding Impact Prefrontal Cortex Activity? An Algorithmic Decoding Approach in Freely Moving Rats
Source: eNeuro. 2018 Apr 27;5(2):ENEURO.0023-18.2018. doi: 10.1523/ENEURO.0023-18.2018 (PMC6192657; doi:10.1523/ENEURO.0023-18.2018)

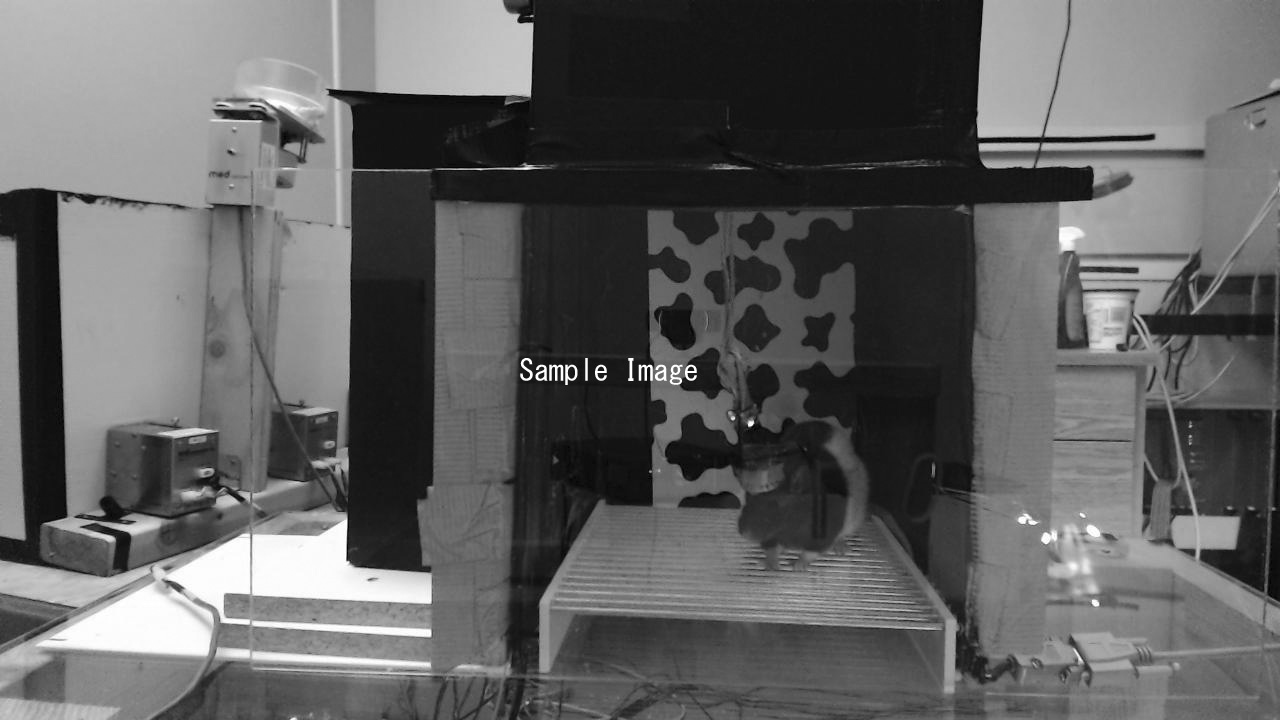

Supplement: Extended Data 1 — Lab video annotator. Compressed code package for the lab video annotator used to annotate video files. Download Extended Data 1, ZIP file. [file sup_enu-eN-NWR-0023-18-s01.zip › Lab_Video_Annotator-master/sampleA.jpg]

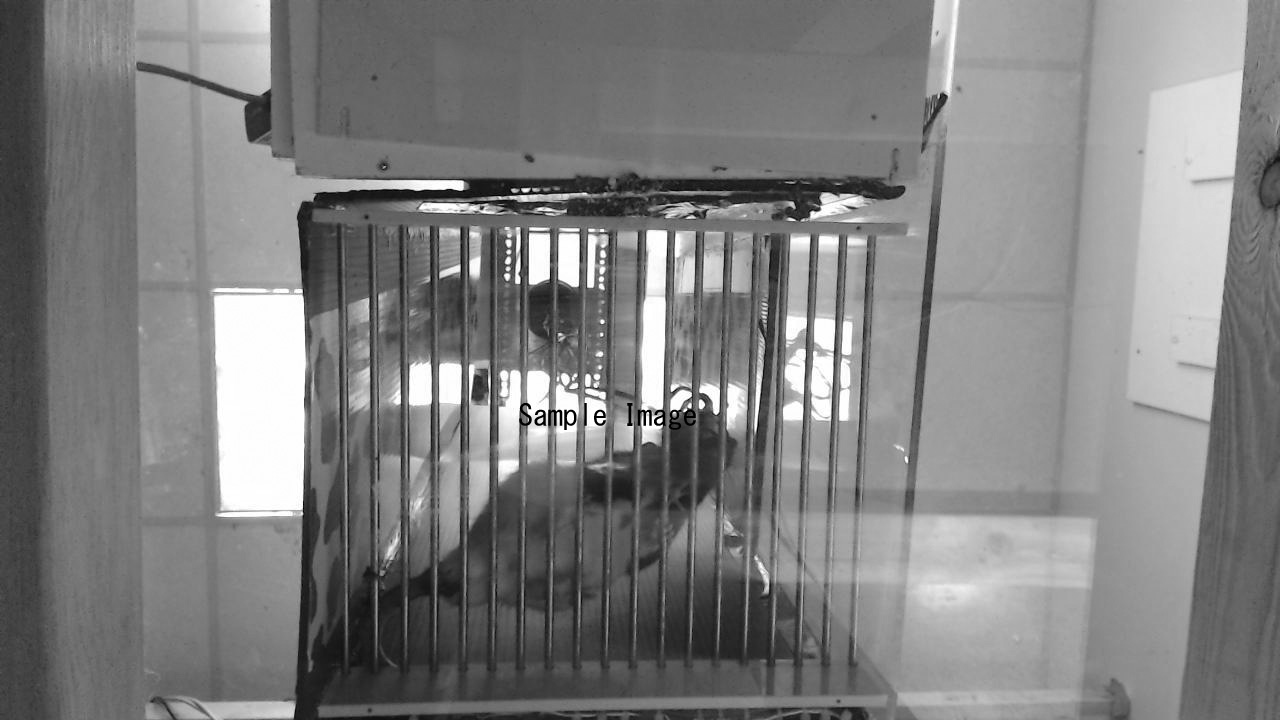

Supplement: Extended Data 1 — Lab video annotator. Compressed code package for the lab video annotator used to annotate video files. Download Extended Data 1, ZIP file. [file sup_enu-eN-NWR-0023-18-s01.zip › Lab_Video_Annotator-master/sampleB.jpg]
